# The impact of COVID‐19 infection on neonatal birth weight and outcomes

**DOI:** 10.1002/pmf2.70137

**Published:** 2025-11-13

**Authors:** Advaita Punjala‐Patel, Sophia Alvarado, Austin French, Rebekah Lassiter, David Stamilio

**Affiliations:** ^1^ Department of Obstetrics and Gynecology Division of Maternal Fetal Medicine Atrium Health Wake Forest Baptist Health Winston Salem North Carolina USA; ^2^ Wake Forest University School of Medicine Winston Salem North Carolina USA

**Keywords:** covid‐19, neonatal birth weight, sga

## Abstract

**Background:**

Since its outbreak in 2019, the novel severe acute respiratory syndrome coronavirus‐2 (COVID‐19) outbreak has had a devastating impact worldwide. Pregnancy has been shown to worsen the clinical outcome in those affected with COVID‐19 infection. While data demonstrate poorer maternal outcomes in those infected with COVID‐19, published studies on neonatal outcomes after antenatal maternal infection are conflicting—specifically as it relates to neonatal birth weight. We hypothesize that maternal COVID‐19 infection increases the risk for small‐for‐gestational age (SGA) neonatal birth weight.

**Objective:**

(1) To estimate the association between antenatal maternal COVID‐19 infection and the risk for SGA and (2) to assess whether gestational timing of infection or severity of COVID‐19 infection differentially impacts the risk of SGA.

**Study design:**

This was a retrospective cohort study that included all pregnant patients with COVID‐19 infection and that randomly selected uninfected controls from October 2020 to June 2022 at a single tertiary care center. Multivariable logistic regression was performed to estimate the independent effect of maternal COVID‐19 infection on SGA neonatal birth weight. We used similar logistical regression models to estimate the independent effect of COVID‐19 exposure on SGA stratified by trimester and COVID‐19 severity.

**Results:**

Of 1011 patients in the study, 502 patients had COVID‐19 infection. Overall, COVID‐19 infection was not associated with an increased risk of SGA, adjusting for confounding by maternal age, hypertension, pre‐gravid body mass index (BMI), and race. However, first trimester infection was associated with a twofold increased risk in SGA (adjusted odds ratio [aOR], 2.3; 95% confidence interval [CI], 1.1–5.1), compared to uninfected controls, while infection in the second and third trimesters was not associated with SGA. Moderate/severe disease was associated with a twofold increased risk of SGA (aOR, 2.1; 95% CI, 1.1–3.5) compared to uninfected controls.

**Conclusion:**

While COVID‐19 infection was not uniformly associated with SGA in the whole cohort, individuals with moderate/severe disease or those exposed in the first trimester had an increased risk of delivering an SGA neonate. Fetal growth surveillance for COVID‐19 infection can be reserved for those with moderate/severe infection or those infected in the first trimester.

## 1 INTRODUCTION

Since its emergence in 2019, COVID‐19 has had catastrophic effects on population health worldwide. Pregnant patients are especially vulnerable, as severe illness seems more common in this population and can be associated with increased maternal morbidity and mortality [1, 2, 3]. The unique immunologic and physiologic alterations associated with pregnancy, including maternal cardiopulmonary changes, predispose pregnant patients to hypoxemic respiratory failure, preeclampsia, and other hypertensive disorders of pregnancy, when infected with the virus [4, 5]. This has led to an increase in critically ill patients admitted to intensive care units with prolonged intubations and adverse long‐term maternal and fetal outcomes. While a few years have passed since the surge of the pandemic, the impact of COVID‐19 on neonatal outcomes remains poorly characterized in the current published literature. Importantly, with the advent of vaccinations, the impact of mild COVID‐19 disease in pregnancy has become a growing topic of interest. Better understanding of the disease and its perinatal effects may help guide providers in counseling and managing patients in the antepartum period [6, 7, 8].

Throughout history, maternal viral infections have been shown to increase the risk of placental inflammatory changes that have led to adverse impact on fetal well‐being. Histopathological tissue examination of placentas demonstrates that COVID‐19 behaves similarly to other viruses and increases risk of maternal vasculature malperfusion by inducing apoptosis and vascular damage [9, 10]. Furthermore, examination of placentas of those affected by COVID‐19 demonstrates higher fibrinoid deposition, a clinical feature of placental injury, when compared to control groups [11, 12, 13]. Initial studies on pregnant individuals have demonstrated that COVID‐19 may increase the risk of abortion, preterm birth, stillbirth, fetal growth restriction (FGR), low birth weight, and neonatal fatality [14, 15, 16]. Studies have suggested that compared with asymptomatic or mild COVID‐19 infection, pregnant individuals with severe illness may be at an increased risk of perinatal complications [17, 18]. It has also been suggested that timing of maternal COVID‐19 infection may be crucial in mediating adverse pregnancy outcomes [19]. Pregnant individuals who were infected with COVID‐19 at the beginning of the gestational period were at higher risk for adverse maternal outcomes, FGR, and preterm delivery, when compared to maternal infection that occurs closer to delivery, presumably due to increased duration of fetal exposure to placental malperfusion [20, 21, 22, 23].

Patients infected with COVID‐19 can exhibit a wide array of clinical symptoms ranging from asymptomatic to critical illness. In the United States, the National Institute of Health (NIH) developed a classification system for grouping patients based on disease severity [24]. According to this classification system, asymptomatic disease is described as an individual who incidentally tested positive for COVID‐19 without any symptoms. Mild disease is described as symptoms of COVID‐19 with signs of fever and cough, but no dyspnea or abnormal chest imaging. Moderate illness is defined as those patients with symptoms of lower respiratory disease or abnormal imaging with SpO_2_ greater than 94% without requiring supplemental oxygenation. Severe illness is defined as dyspnea with oxygen requirements, respiratory failure, or abnormal imaging demonstrating profuse lung infiltrations affecting greater than 50% of the lung fields [11, 24, 25, 26].

Data on neonatal outcomes based on COVID‐19 severity and trimester of infection remain ill‐defined and sparse. Reviews have noted an effect of COVID‐19 infection on pregnancy outcomes and birth outcomes, like stillbirth and preterm delivery; however, little data exist on small‐for‐gestational age (SGA) [27, 28, 29]. Given these knowledge limitations, evidence‐based guidelines for routine ultrasound surveillance of fetal growth or fetal antenatal testing after COVID‐19 infection cannot be developed.

The primary aim of this study is to estimate the association between maternal COVID‐19 infection and the risk of delivering an SGA neonate, defined as neonatal birth weight less than 10th percentile for gestational age. Furthermore, we aim to assess whether there is an association between COVID‐19 infection severity as defined by the NIH classification [11, 25, 26] or trimester‐specific timing of maternal COVID‐19 infection and SGA. We hypothesize that maternal COVID‐19 infection independently increases the risk for SGA neonatal birth weight.

## 2 MATERIALS AND METHODS

### 2.1 Study design and population

We conducted a retrospective cohort study of all patients with a COVID‐19 diagnosis and randomly selected uninfected controls who delivered at Atrium Health Wake Forest Baptist Medical Center from October 1, 2020 to June 1, 2022. Wake Forest Baptist Medical Center is a tertiary care university‐based medical center that delivers approximately 3200 pregnant patients a year. Our cohort of patients included pregnant individuals who are older than 18 years, with non‐anomalous singleton fetuses. Pregnancies complicated by fetal anomalies were excluded from our study due to anticipated effects on birth weight, our primary outcome. The diagnosis of COVID‐19 infection was made following a positive RT‐PCR (reverse transcription polymerase chain reaction) naso‐oropharyngeal swab test analyzed at any time during the patient's antenatal course. Patients with a diagnosis of antenatal COVID‐19 infection were identified by a combination of a positive RT‐PCR test for COVID infection or International classification of disease (ICD)‐10 codes U07.1/U07.2. A thorough chart review was performed to validate the diagnosis and identify the timing of the infection as it related to gestational age.

Unexposed control patients without antenatal COVID‐19 infection were selected using a random number generator from the cohort of patients that delivered during the study period. January, 2020 was chosen as the start date of this cohort population because this date coincided with when COVID‐19 RT‐PCR tests were readily available in our hospital and when we were performing universal screening for COVID‐19 in all pregnant patients seen in or admitted to our Labor and Delivery unit, OB triage unit, any medical unit, or emergency department, regardless of symptoms. We chose to end the study on June 1, 2022 because this was the date that the institution stopped universal testing for pregnant patients admitted to the Labor and Delivery unit. Thus, this time period was optimal for minimizing misclassification bias regarding COVID‐19 exposure.

This study was approved by Atrium Health Wake Forest Institutional Review Board (IRB No. 00090379). Maternal and neonatal data were abstracted through manual electronic medical record review (EMR) using a standardized data collection form with detailed and specific variable definitions. Study data were collected and managed in a secure research electronic database (REDCAP). Other variables extracted included demographic data on antepartum COVID‐19 vaccination status, and antenatal co‐morbidities such as diabetes or hypertension. Antepartum COVID‐19 vaccination receipt was defined as those patients who received at least the first dose of any available vaccine during the study period. COVID‐19 vaccination status was determined by a combination of patient self‐report and EMR searching for an uploaded Centers for Disease Control and Prevention (CDC) COVID‐19 vaccination card or record of the vaccine in the drug administration section. Diabetes was defined as pregestational or gestational. Pregestational diabetes included Type I or Type II Diabetes Mellitus, defined by the American Diabetic Association [30]. Gestational diabetes was defined as any degree of glucose intolerance as identified by a 50 g, 1 h oral glucose tolerance test and confirmed with a 100 g, 3 h glucose [31]. Hypertensive disorders of pregnancy were defined per standard American College of Obstetricians and Gynecologists guidelines [32, 33]. For purposes of analysis, patients with any type of gestational hypertension (pregnancy‐induced hypertension) were categorized as gestational hypertension or preeclampsia; thus, the chronic hypertension category represents patients with chronic hypertension without gestational hypertension.

### 2.2 Outcomes

The primary outcome was delivery of an SGA neonate defined by a neonatal birth weight of less than 10th percentile based on the World Health Organization (WHO) growth curve, utilized if gestational age at delivery was greater than or equal to 37 0/7 weeks of gestation [34]. In neonates born between 22 0/7 weeks and 37 0/7 weeks of gestation, SGA was defined as less than the 10th percentile based on the Fenton curve [35]. These curves are validated neonatal growth curves used at our institution to describe neonatal birth weights based on gender as assigned at birth. Gestational age at birth was calculated from the expected due date (EDD) based on the best obstetric estimate as defined by American College of Obstetrics and Gynecology (ACOG) [36].

The secondary outcomes included neonatal birth length percentile, neonatal head circumference percentile, neonatal intensive care unit (NICU) admission as a dichotomous variable, and neonatal hospitalization length of stay. The other neonatal biometric data were evaluated on the same growth curves used to assess birth weights based on neonatal gestational age at delivery [34, 35]. Gestational timing was categorized by trimester of infection using standard definitions by ACOG [37]. Severity of COVID‐19 infection was based on NIH classification and classified into three groups [24]—asymptomatic/mild infection, moderate infection, and severe infection. Asymptomatic patients were those who incidentally tested positive for COVID‐19 upon admission to Labor and Delivery unit on universal screening or any time during their antenatal course, and mild infection patients were those who had signs and symptoms of COVID‐19 but no shortness of breath or abnormal chest imaging. Moderate illness was defined as those patients with abnormal chest imaging or signs of lower respiratory disease with SpO_2_>94%. Severe illness was defined as those patients who had oxygen requirements with SpO_2_ < 94% at room air, respiratory frequency>30 breaths/min or lung infiltrates in>50% of lung fields on imaging. The severe infection group also included patients with signs of respiratory failure, admission to intensive care unit, septic shock, and signs concerning for multiple organ dysfunction. For purposes of statistical analyses, we combined moderate and severe COVID‐19 disease into a single category because of the low frequency of severe disease in our population.

### 2.3 Statistical analysis

We performed the analysis using STATA software (version 15.0). Descriptive statistics were utilized to compare demographic data and clinical co‐morbid conditions between the COVID‐19 infection exposed and unexposed groups.

The Mann–Whitney *U* test or Student's *t* test was used for continuous variables depending on whether the data appeared to be normally distributed by the Shapiro Wilk test, and chi‐square test was used for categorical variables. In the primary analysis, COVID‐19 was analyzed as a dichotomous exposure variable. In a planned secondary analysis, we assessed COVID‐19 exposure as a multi‐categorical exposure variable for disease severity (unexposed, asymptomatic/mild infection, or moderate/severe infection) and gestational timing of infection (unexposed, first trimester, second trimester, or third trimester) since it is biologically plausible that severe or earlier disease may have a greater impact on the primary outcome. We used multivariable logistic regression to estimate the adjusted odds ratio (aOR) and 95% confidence interval (CI) for the primary exposure‐outcome association. Candidate variables for multivariable models were selected based on the unadjusted analysis of baseline characteristics and based on historically important factors identified in prior research. The candidate variables included as potential confounding factors in the initial multivariable models included maternal age, parity, prepregnancy body mass index (BMI), chronic hypertension, gestational hypertension/preeclampsia, diabetes mellitus, health insurance type, vaccination status, and self‐reported maternal race and ethnicity. Only covariates that were significantly associated with the outcome and appreciably changed the adjusted effect size (> 10% change in OR) for the COVID‐SGA association were retained in the final regression models. The final logistic regression models included hypertensive disorders, prepregnancy BMI, and maternal race and ethnicity. For purposes of results description, we consider the aOR as a reasonably accurate estimate of relative risk (RR) since the rate of the primary outcome is<10%. Because there is concern that vaccination may modify the effect of infection on pregnancy outcomes, we performed an exploratory analysis of the effect of COVID‐19 infection, infection gestational timing, and infection severity on the risk of SGA stratified by maternal COVID‐19 vaccination status during the pregnancy and prior to COVID‐19 infection in exposed patients. We had complete data for all variables except for BMI (9 missing), hypertension (1 missing), gestational timing of COVID19 infection (1 missing), and COVID vaccine status (51 missing); all rates of missingness were<1% except vaccine status which was 5%. Since there is minimal missing data and data are complete for primary exposure and outcome variables, we did not apply data imputation methods. All statistical tests were two sided, and *p *< 0.05 was considered statistically significant. We did not adjust alpha error for multiple comparisons for the secondary outcomes.

### 2.4 Sample size calculation

Our a priori sample size calculations assumed a baseline risk for SGA neonates to be approximately 10%. We planned equally sized COVID‐19 exposed and unexposed groups (1:1 ratio). With alpha error of 0.05, and power of 80%, we estimated needing 200 patients in each group (total *N* = 400) to detect a twofold increase in neonatal SGA birth weight among those with maternal COVID‐19 disease compared to those without. However, to estimate risks stratified by trimester of exposure and to estimate the effect of COVID‐19 severity levels we estimated requiring 400 patients in each group (total *N* = 800) to detect a twofold increase (RR = 2) in neonatal SGA birth weight among the groups of early versus late gestational exposure compared to no COVID‐19 infection. This estimate is based on empiric unpublished data from our institution that suggest about 50% of patients experience COVID‐19 early in pregnancy (defined as first and second trimesters) and 50% are infected in the third trimester. Data from our institution also indicate that only 10% of COVID‐19 cases are severe, defined as requiring maternal intensive care unit admission. Thus, we anticipated 40 patients with severe disease to be in this cohort. Based on an anticipated 10% rate of severe disease, we estimated that this study would be adequately powered (80%) to detect a 2.6‐fold increase in the primary outcome (SGA) compared to no exposure or lesser disease severity.

## 3 RESULTS

Among 1011 patients included in this cohort study, 502 patients had antenatal COVID‐19 infection, which represent approximately 9% of the total birth cohort (*N* ∼ 5400). The unexposed group is slightly larger than the COVID‐19 exposed group after exclusion criteria were applied. Baseline characteristics between the COVID‐19 exposed group and the unexposed group are described in Table 1. All patients who were diet controlled or on pharmacological treatment for gestational diabetes were characterized into a common demographic group for the purpose of the table. For most factors, the baseline characteristics between our two study groups were similar; however, the group without COVID‐19 infection had a higher proportion of patients affected with hypertensive disorders of pregnancy. The patients in our control group also had a statistically significant higher parity. Unsurprisingly, the control group without COVID‐19 infection had a higher proportion of patients who received at least one dose COVID‐19 vaccine antenatally. Interestingly, patients without COVID‐19 infection had a higher rate of successful vaginal delivery including vaginal births after cesareans (VBAC) compared to those patients infected with COVID‐19. There was also one planned cesarean hysterectomy in the COVID‐19 infected group for sonographic findings consistent with placenta accreta spectrum.

**TABLE 1:** Study demographics.

	COVID‐19 uninfected (*n* = 509)	COVID‐19 infected (*n* = 502)	*p* value
Age^a^ (years)	28 ± 9	29 ± 8	0.43
Prepregnancy BMI^a^ (kg/m^2^)	31 ± 11.3	30.1 ± 9.5	0.09
Gestational age at delivery^b^ (weeks)	39.1 (37.1–39.4)	39.0 (37.5–38.6)	0.51
Race^c^			0.82
White	49.3% (251)	47.4% (238)	
African American	21.4 % (109)	22.1% (111)	
Asian	2.2 % (11)	2.6% (13)	
Latin American	6.9% (35)	9.2% (46)	
Other	20.2% (103)	18.7% (94)	
Ethnicity^c^			0.90
Not Hispanic	74.5% (379)	75.7% (380)	
Hispanic	25.3% (129)	24.1% (121)	
Unknown	0.2% (1)	0.2% (1)	
Parity^b^	2 (1–2)	1 (0–2)	<0.001
Insurance^c^			0.04
Self‐pay	7.7% (39)	12.5% (63)	
Medicaid	47.0% (239)	45.8% (230)	
Private	45.4% (231)	41.6% (209)	
COVID vaccine^c^			0.001
Never	64.8% (330)	75.5% (379)	
Received	29.7% (151)	19.7% (99)	
Unknown	5.5% (28)	4.8% (24)	
Smoking^c^			0.16
Never	86.1% (438)	81.5% (409)	
Past	7.7% (39)	10.6% (53)	
Current	6.3% (32)	7.9% (40)	
Diabetes^c^			0.95
Pregestational	10.6% (54)	5.2% (26)	
Gestational	14.1% (72)	7.4% (37)	
Hypertension^c^			0.001
Chronic	15.9% (81)	7.2% (36)	
Gestational	9.4% (48)	8.2% (41)	
Preeclampsia/HELLP/eclampsia	25.9%(132)	8.2% (41)	
Delivery mode^c^			<0.001
Vaginal	66.6% (339)	58.4% (293)	
Cesarean	29.0% (148)	37.8% (190)	
Operative vaginal	2.2% (11)	1.6% (8)	
VBAC	2.2% (11)	2.0% (10)	
APGAR scores^b^ (5 min)	8 (7–8)	8 (8–9)	0.23

Abbreviations: BMI, body mass index; HELLP, Hemolysis, elevated liver enzymes, low platelets; VBAC, vaginal births after cesareans.

^a^ Data are shown as mean ± standard deviation.

^b^ Median (interquartile range).

^c^ Number (percentage).

The rates of SGA birth weight were 9.6% and 7.3% in the COVID‐19 infected group and the uninfected control group, respectively (Table 2). In the unadjusted analysis of the primary outcome, the rate of SGA was similar between COVID‐19 exposed and unexposed groups (unadjusted RR, 1.3; 95% CI, 0.9–1.9; *p* = 0.52). The final logistic regression model adjusting for confounding factors (hypertension, prepregnancy BMI, and race/ethnicity) confirmed no significant association between maternal COVID‐19 infection and SGA, with similar effect size (Table 2).

**TABLE 2:** Impact of COVID‐19 infection on small‐for‐gestational age (SGA) and other secondary outcomes.

	COVID‐19 uninfected (*n* = 509)	COVID‐19 infected (*n* = 502)	Unadjusted RR (95% CI)	Adjusted OR (95% CI)
SGA^a^	7.3% (37)	9.6% (48)	1.3 (0.9–1.9), *p *= 0.52	1.3 (0.9–2.1), *p *= 0.52

Secondary outcomes	COVID‐19 uninfected (*n* = 509)	COVID‐19 infected (*n* = 502)	*p* value
NICU admission^a^	16.5% (84)	18.5% (93)	0.39
Neonatal LOS^b^, ^[Link]^ (days)	4.1 ± 12.1	4.6 ± 11.3	0.18
Neonatal head circumference^b^ (percentile)	49.0 ± 32.2	52.6 ± 11.3	0.07
Neonatal birth length^b^ (percentile)	64.5 ± 31.4	64.6 ± 30.3	0.53

Abbreviations: BMI, body mass index; CI, confidence interval; LOS, length of neonatal stay in hospital; NICU, neonatal intensive care unit; OR, odds ratio; RR, relative risk.

^a^ Data are reported as percentage (number). For NICU admission, data is dichotomous and represents the percentage (number) of neonates admitted to the NICU after birth.

^b^ Mean ± standard deviation.

^*^Logistic regression models adjusted for hypertensive disorders, prepregnancy BMI, and maternal race and ethnicity.

Next, we assessed antenatal COVID‐19 infection exposure in terms of gestational timing of infection and clinical disease severity. Among 502 maternal patients with COVID‐19 infection, 15.2% (76) had infection in the first trimester, 31.1% (156) had infection in the second trimester, and 53.4% (270) had infection in the third trimester. Stratifying by gestational timing of COVID‐19 infection, 13.0% (10) had neonatal SGA with first trimester infection exposure, 10.8% (17) with second trimester exposure, and 7.8% (21) with third trimester exposure. The rate of SGA in pregnant individuals without prenatal COVID‐19 infection was 7.3% (37), which is similar to the rate seen in patients with third trimester COVID‐19 infection. In multivariable analysis, first trimester maternal COVID‐19 infection was associated with a twofold increased risk of delivering an SGA neonate (aOR, 2.3; 95% CI, 1.1–5.1; *p* = 0.03) compared to the unexposed group. While the observed rate of SGA in second trimester infection was 1.5‐fold higher than the unexposed group, this difference was not statistically significant (*p* = 0.18). Multivariable analysis also confirmed that the odds of SGA is similar between those with third trimester infection and those without infection (Table 3).

**TABLE 3:** Impact of COVID‐19 infection by trimester of infection on SGA.

Trimester of infection	SGA neonate (%; *n*)	Unadjusted RR (95% CI)	Adjusted OR (95% CI)	*p* value
Control (no infection)	7.3% (37)	Reference	Reference	Reference
First trimester (*n* = 76)	13.0% (10)	2.1 (1.1–4.1)	2.3 (1.1–5.1)	0.03^*^
Second trimester (*n* = 156)	10.8% (17)	1.5 (0.8–2.6)	1.5 (0.8–2.9)	0.18
Third trimester (*n* = 270)	7.8% (21)	1.0 (0.6–1.7)	1.0 (0.6–1.8)	0.92

*Note*: Data are reported as percentage (number).

Abbreviations: BMI, body mass index; CI, confidence interval; OR, odds ratio; RR, relative risk; SGA, small‐for‐gestational age.

* Logistic regression models adjusted for hypertensive disorders, prepregnancy BMI, and maternal race and ethnicity.

Stratifying by maternal COVID‐19 disease severity, 68.4% (343) had asymptomatic/mild infection, 25.4% (128) had moderate infection, and 6.2% (31) had severe infection. Because of the low frequency of patients with clinically severe infection, we combined patients with moderate and severe disease into a single stratum for the analysis to improve precision of estimates. The rate of neonatal SGA in those affected with asymptomatic or mild COVID‐19 infection was 7.8% (27), and the rate of SGA in those with moderate/severe disease was 13.3% (21); while the rate of having an SGA neonate in those without COVID‐19 infection was 7.3% (37). Adjusting for the same covariates as the above regression models, moderate‐to‐severe disease was associated with a twofold increased risk of having an SGA neonate compared to the unexposed group (aOR, 2.1; 95% CI, 1.2–3.8; *p* = 0.014), but asymptomatic/mild disease was not associated with an increased odds for delivering an SGA neonate (Table 4). The distribution of disease severity level was similar across all three trimesters (*p* = 0.52), indicating that disease severity is unlikely a confounder of the effect of timing of infection on SGA (Table 5).

**TABLE 4:** Impact of COVID‐19 infection by severity on SGA.

COVID‐19 infection severity	SGA neonate (%; *n*)	Unadjusted RR (95% CI)	Adjusted OR (95% CI)	*p* value
Control (no infection)	7.3% (37)	Reference	Reference	Reference
Asymptomatic/mild disease (*n* = 343)	7.8% (27)	1.0 (0.5–1.7)	1.1 (0.6–1.8)	0.84
Moderate/severe disease (*n* = 159)	13.3% (21)	2.2 (1.1–3.5)	2.1 (1.2–3.8)	0.01^*^

*Note*: Data are reported as percentage (number).

Abbreviations: BMI, body mass index; CI, confidence interval; OR, odds ratio; RR, relative risk; SGA, small‐for‐gestational age.

* Logistic regression models adjusted for hypertensive disorders, prepregnancy BMI, and maternal race and ethnicity.

**TABLE 5:** COVID‐19 infection severity by trimester of infection.

COVID‐19 infection severity	First trimester infection	Second trimester infection	Third trimester infection	*p* value
Asymptomatic/mild disease (*n* = 343)	69.7% (53)	64.7%% (101)	70.0% (189)	0.52
Moderate/severe disease (*n* = 159)	30.3% (23)	35.3% (55)	30.0% (81)	

*Note*: Data are reported as percentage (number).

Maternal COVID‐19 infection when assessed as a dichotomous exposure variable was not associated with an increased rate of any secondary outcomes, including neonatal length of stay (days), NICU admission, neonatal head circumference percentile at birth, and neonatal birth length percentile (Table 2). When assessing COVID‐19 exposure by trimester of infection and by disease severity, COVID‐19 infection was again not associated with an increased risk of any of the secondary outcomes (Table 6). Of note, these secondary outcomes were exploratory in nature, and therefore *p* values and confidence intervals are given for descriptive purposes only as no adjustments for multiple comparisons were made.

**TABLE 6:** Secondary outcomes by trimester and severity of COVID‐19 infection.

Secondary outcomes	First trimester	Second trimester	Third trimester	*p* value
Neonatal LOS (days)	4.2 ± 12.3	3.9 ± 8.1	3.1 ± 10.1	0.65
Neonatal head circumference (percentile)	50.4 ± 34.1	53.7 ± 32.7	53.4 ± 31.5	0.68
Neonatal birth length (percentile)	65.2 ± 32.1	64.4 ± 29.4	64.3 ± 30.6	0.73
NICU admission^a^	5.7% (10)	18.1% (32)	29.2% (52)	0.44

	Asymptomatic/mild infection	Moderate/severe infection	*p* value
Neonatal LOS (days)	4.2 ± 12.5	4.9 ± 8.3	0.66
Neonatal head circumference (percentile)	52.4 ± 31.5	53.5 ± 33.1	0.79
Neonatal birth length (percentile)	63.8 ± 30.2	66.2 ± 30.7	0.77
NICU admission^a^	34.5% (61)	18.1% (32)	0.57

*Note*: Data reported as mean ± standard deviation.

Abbreviations: LOS, length of neonatal stay in hospital; NICU, neonatal intensive care unit.

^a^ For NICU admission, data are dichotomous and represent the percentage (number) of neonates admitted to the NICU after birth.

Because there is some concern that maternal prior COVID‐19 immunity may modify the effect of COVID‐19 on SGA, we performed an analysis of COVID‐19 infection, infection gestational timing (trimester), and infection severity stratified by maternal COVID‐19 vaccination status during pregnancy. While the stratification reduces sample size and consequently effect estimate precision, the rates and aOR point estimates for SGA are generally similar to the overall analysis and similar between the vaccinated and unvaccinated strata with the same direction of effect and largely comparable effect size (Tables S1–S3). Confidence intervals are wide, particularly for the smaller vaccinated population, but the effect of moderate‐to‐severe COVID‐19 disease remained statistically significant with an aOR of 2.1 (95% CI, 1.1–4.3).

## 4 COMMENTS

### 4.1 Principal findings

In this cohort, maternal COVID‐19 infection overall did not appear to increase the risk of delivering an SGA neonate; however, when stratifying by disease severity or trimester of infection, we observed an increased risk of SGA birth weight in those patients affected with COVID‐19 infection in the first trimester or with moderate‐to‐severe COVID‐19 infection in any trimester.

### 4.2 Results in the context of what is known

Pregnant individuals are among the most vulnerable to be affected by adverse health outcomes secondary to COVID‐19 infection and much remains to be studied on the effects of antenatal infection and perinatal outcomes. Neonatal birth weight is a sensitive indicator of intrauterine growth and can be an important predictor of overall neonatal health [38]. In our study, we chose to investigate SGA as opposed to FGR to limit the risk of selection bias due to confounding by indication for having a fetal growth ultrasound. During the pandemic, all patients in our health system who tested positive for antepartum COVID‐19 infection, regardless of severity or timing of infection, had serial fetal growth ultrasounds to screen for FGR. In patients without antenatal COVID‐19 infection, fetal growth ultrasounds were reserved only for those who had an indication for antenatal fetal surveillance, such as hypertension or diabetes [39]. Low‐risk patients without COVID‐19 infection did not undergo routine fetal growth surveillance. Thus, studying FGR in this retrospective cohort would have significant risk of selection bias since low‐risk patients (those without a clinical indication for fetal ultrasound) would be underrepresented in the control group. The selection bias would likely falsely increase the estimated rate of FGR in the control group, masking or diminishing the association between COVID‐19 and FGR.

To our knowledge, this is the first cohort study to investigate the effect of antenatal COVID‐19 severity and timing of infection on neonatal risk of SGA birth weight. A recent meta‐analysis of 4514 studies across a large population from 22 countries compared mean birth weight during the pandemic to the pre‐pandemic period, and similar to our study did not find an overall difference in the rate of SGA in those affected with COVID‐19 infection; however, this study did not account for severity or timing of infection. Furthermore, this meta‐analysis investigated pre‐pandemic and post‐pandemic timing, specifically isolating the impact of lockdown on neonatal growth, as opposed to antenatal COVID‐19 infection. While the study population in this meta‐analysis was large, there is also an increased risk of bias associated when investigating two different time periods since multiple time‐related factors may impact SGA risk [40].

Another meta‐analysis of 10 studies demonstrated findings similar to our study with a higher risk of SGA neonates in those patients affected by symptomatic COVID‐19 disease as opposed to asymptomatic disease; however, unlike our study, symptomatic COVID‐19 patients were compared to those with asymptomatic or mild disease instead of COVID‐19 negative controls, precluding an assessment of the full effect of COVID‐19 infection relative to those without infection [41]. Only one cohort study has shown an increased risk of FGR in severe disease; however, this study also uses mild/asymptomatic COVID‐19 infection as the control group, as opposed to those uninfected by COVID‐19 infection, increasing the risk of bias [22]. In addition, prior cohort studies assessing neonatal SGA risk after COVID‐19 infection have only compared trimester of infection to asymptomatic infected control groups and, unfortunately, may not have been powered to estimate a difference between trimester of COVID‐19 infection and uninfected controls [42]

Past research has suggested that COVID‐19 infection has the potential to negatively impact pregnancy and neonatal outcomes [1, 14, 15]. Other viral infections, such as cytomegalovirus, influenza, rubella, and zika, have been shown to increase the risk of adverse perinatal outcomes including FGR, spontaneous abortion, preterm birth, and other adverse neonatal sequelae, likely by exploiting the immune‐modulated state of pregnancy and mediated by disrupting the placental‐fetal interface or direct fetal affect [43]. Animal models have shown that COVID‐19 has a unique spike‐like protein on its surface that must bind to angiotensin receptor enzyme (ACE‐2) receptors to replicate and increase its pathogenic potential in the human body [44]. ACE‐2 receptors are metalloenzymes also found in the cell membranes of various organs including the lungs, heart, kidneys, and intestines, but importantly, these receptors are abundantly expressed in the placenta and other reproductive organs. The ACE‐2 enzyme in the human placenta is expressed by native placental cells including cytotrophoblasts, synctiotrophoblasts, extravilloustrophoblasts (EVTs), and endothelial cells [44, 45].

Increased ACE‐2 expression in placental tissue is hypothesized to be at least partly responsible for placental infection by COVID‐19, increasing the risk of placental dysfunction [46]. The rationale for assessing gestational timing of maternal COVID‐19 infection as a factor in the pathogenesis of SGA birth weight stems from the hypothesis that COVID‐19 may target the ACE‐2 in the placenta increasing the risk of placental insufficiency. Hypothetically, earlier maternal infection compared to third trimester infection prolongs the period of placental exposure to the virus and its inflammatory effects and increases the duration of risk for placental injury, which in turn could increase the risk of FGR and SGA birth weight. Similarly, it is plausible that a more severe COVID‐19 infection could predispose an individual to increased thrombotic inflammatory activation and inadequate uteroplacental perfusion due to the upregulation of ACE‐2, increasing the risk of low birth weight [44, 46, 47].

### 4.3 Clinical implications

SGA and FGR are short‐term indicators of neonatal and fetal health, respectively, which can increase risk for adverse perinatal outcomes [38]. Our study findings demonstrate that there is an increased odds of SGA associated with first trimester COVID‐19 infection or moderate/severe disease in any trimester. Based on the findings of our study, we recommend considering fetal growth ultrasounds in individuals with prenatal COVID‐19 infection if they experience clinically moderate‐to‐severe disease or if they were infected with COVID‐19 in the first trimester irrespective of disease severity. The findings of our study also suggest the potential maternal benefits of the COVID‐19 vaccination as a larger proportion of patients who were infected with COVID‐19 were unvaccinated.

Because the majority of this cohort (75%) did not receive the COVID‐19 vaccination and because this cohort was developed early in the pandemic, one may question if these results are generalizable to a largely vaccinated population or a contemporary population with higher rates of immunity from prior disease and/or vaccination. To explore a variable effect of COVID‐19 infection due to pre‐existing immunity, we performed an analysis of the primary exposure‐outcome associations stratified on whether patients had received a COVID_19 vaccination. While the resulting stratum‐specific sample sizes were relatively small, precluding definitive conclusions, the results of this exploratory analysis suggest that the effect of infection early in gestation and of moderate‐to‐severe disease appears to be stable across those who did or did not receive a vaccine since OR point estimates are similar, albeit with rather wide 95% CIs. In addition, we believe that these results may be applicable to contemporary obstetric populations for three reasons. First, the current estimated rate of COVID‐19 vaccination during pregnancy is only 14.4% based on 2024–2025 CDC data [48]; therefore, most pregnant patients have not been vaccinated recently. Second, immunity is known to wane over time, and importantly in the context of low vaccination rates, the immunity conferred by infection compared to vaccine is less durable [49, 50]. Third, as new COVID‐19 variants emerge, distant prior vaccines are likely to have reduced efficacy for disease prevention [49]. Based on the above vaccination and disease trends and our exploratory analysis, and until a contemporary cohort with COVID‐19 immunity can be analyzed, we suggest that these results and consideration for fetal growth ultrasounds in individuals with clinically moderate‐to‐severe COVID‐19 disease or disease in the first trimester be generalized to the population of pregnant patients irrespective of prior immune status.

### 4.4 Research implications

This is one of the first studies to specifically investigate the impact of COVID‐19 infection on the risk of delivering an SGA neonate, stratified by trimester and severity of infection. Future studies that include a larger sample of patients with severe COVID‐19 disease would help better characterize the differential effect on neonatal outcomes between moderate and severe clinical disease. Furthermore, little is known about whether maternal vaccination or maternal monoclonal antibodies for treatment of COVID‐19 in the prenatal period has a protective effect for delivering an SGA neonate or for other adverse neonatal outcomes. These interventions should be a focus of future research.

### 4.5 Strengths and limitations

A strength of our study was our institution's opportune use of universal COVID‐19 infection RT‐PCR testing at the time of admission to Labor and Delivery unit or antepartum floor during the cohort period. In addition, institutional policy also promoted liberal use of COVID‐19 RT‐PCR testing in the emergency department and obstetric triage area. This policy allowed us to minimize misclassification bias regarding exposure since without these screening policies patients with asymptomatic or mild disease at admission may be misclassified as unexposed. However, exposure status misclassification cannot be avoided completely. There is a chance that residual misclassification bias may exist since universal screening was only performed in the hospital, and patients without symptoms or with mild symptoms may not have sought COVID‐19 testing. In this scenario, patients with COVID‐19 infection with no or minimal symptoms may have been included in the unexposed control group. Such misclassification bias would diminish the observed magnitude of the effect of COVID‐19 on SGA risk since the unexposed group would incorrectly contain some ill patients. Therefore, if significant disease misclassification bias were at play, COVID19‐related risks might be underestimated. There is also the possibility of misclassification of COVID‐19 vaccination status as some patients received vaccination outside of our health system (e.g., an external pharmacy). However, we believe that risk for significant misclassification bias is minimal as vaccine documentation was prioritized and was templated in the EMR during this period of the pandemic.

Other strengths include rigorous data collection and management processes. To decrease the risk of potential misclassification bias for COVID severity based solely on ICD‐10 codes, data from each medical record were manually extracted and judiciously interpreted by our team based on the strict NIH criteria. A second abstractor assessed a subset of variables and discrepancies were resolved through consensus. We developed strict and distinct definitions for exposure and outcome variables, as well as for covariates, which helps minimize information bias. We had a low frequency of missing data decreasing risk of follow‐up bias. Finally, we used multivariable analytic methods to minimize residual confounding of the association between COVID‐19 infection and SGA.

Our study's primary limitation is the retrospective observational study design, which limits our ability to definitively establish a causal relationship between COVID‐19 and SGA birth weight. However, the fact that we observed a relationship between severity of COVID‐19 disease and SGA, as well as a stronger effect on SGA with infection earlier in the gestation helps support the notion of a causal relationship since both findings are concordant with biologic plausibility. Additionally, this study was conducted at a single academic referral center, which may limit the generalizability of the findings, particularly to lower‐risk obstetric populations. As alluded to previously, the small sample size of patients with severe disease in this cohort restricts our ability to draw definitive conclusions about this subgroup. Similarly, the 95% CIs around the SGA aORs for first trimester infection and moderate/severe infection are relatively wide, indicating that a larger study would provide more precision and therefore more confidence in the estimates.

## 5 CONCLUSIONS

In this cohort of pregnant people, although maternal COVID‐19 infection does not uniformly increase SGA risk in unselected cases of infection, those infected with antepartum COVID‐19 in the first trimester or those with moderate‐to‐severe disease in any trimester had an increased risk of delivering an SGA neonate. Additional studies are needed to validate these findings in other obstetric populations. However, until further research is available, our findings suggest that for patients with moderate‐to‐severe COVID‐19 disease or early prenatal COVID‐19 infection of any severity level, obstetric providers should consider offering third trimester ultrasounds to screen for FGR, which, if detected, would prompt antenatal fetal testing to reduce perinatal morbidity and mortality.

## CONFLICT OF INTEREST STATEMENT

The authors declare no conflicts of interest.

## FUNDING INFORMATION

The authors received no specific funding for this work.

## Supporting information



Supporting Information

## Data Availability

The data that support the findings of this study are available from the corresponding author upon reasonable request.
