# Supplementary material for: The impact of COVID‐19 infection on neonatal birth weight and outcomes
Source: Pregnancy (Hoboken). 2025 Nov 13;1(6):e70137. doi: 10.1002/pmf2.70137 (PMC13344791; doi:10.1002/pmf2.70137)
Supplement: Supplementary file 1 [file PMF2-1-e70137-s001.docx]

***Supplemental Table 1:* Impact of COVID-19 Infection on Small-for-Gestational Age (SGA) stratified by COVID-19 vaccination status**

|  | **COVID-19 uninfected** | **COVID-19 infected** | **Unadjusted RR (95% CI)** | **Adjusted OR (95% CI)** |
| --- | --- | --- | --- | --- |
| **SGA* among vaccinated (N=249)** | 6.6% (10/151) | 11.2% (11/98) | 1.7 (0.7-3.8) | 1.6 (0.6-4.1) |
| **SGA* among Unvaccinated (N=711)** | 7.2% (24/330) | 8.9% (34/381) | 1.2 (0.7-2.0) | 1.3 (0.7-2.3) |

*Data are reported as percentage (number/denominator). The logistic regression models include the same covariates as the models for the overall cohort. Vaccine status is unknown for 51 subjects (5%).

***Supplemental Table 2:* Impact of COVID-19 infection by Trimester of Infection on SGA stratified by COVID-19 vaccination status**

| **Trimester of infection** | **SGA neonate %(n/d)** | **Unadjusted OR (95% CI)** | **Adjusted OR (95% CI)** |
| --- | --- | --- | --- |
| **Vaccinated population (N=249)** | | | |
| No infection (n=151) | 6.6% (10) | Reference | Reference |
| First Trimester  (n= 10) | 20.0% (2) | 3.5 (0.7-18.9) | 5.1 (0.8-30.4) |
| Second Trimester  (n= 33) | 15.2% (5) | 2.5 (0.8-7.9) | 2.6 (0.8-8.8) |
| Third Trimester  (n= 54) | 7.4% (4) | 1.1 (0.3-3.8) | 0.9 (0.3-3.1) |
| **Unvaccinated population (N=711)** | | | |
| No infection (n=330) | 7.3% (24) | Reference | Reference |
| First Trimester  (n= 63) | 9.5% (6) | 1.3 (0.5-3.4) | 1.6 (0.6-4.3) |
| Second Trimester  (n= 114) | 9.7% (11) | 1.4 (0.6-2.9) | 1.3 (0.6-2.9) |
| Third Trimester  (n= 204) | 8.3% (17) | 1.2 (0.6-2.2) | 1.2 (0.6-2.3) |

The logistic regression models include the same covariates as the models for the overall cohort. Vaccine status is unknown for 51 subjects (5%).

***Supplemental Table 3:* Impact of COVID-19 Infection by Severity on SGA stratified by COVID-19 vaccination status**

| **COVID-19 Infection Severity** | **SGA neonate (%; n)** | **Unadjusted OR (95% CI)** | **Adjusted OR (95% CI)** |
| --- | --- | --- | --- |
| **Vaccinated population (N=249)** | | | |
| No infection (n=151) | 6.6% (10) | Reference | Reference |
| Asymptomatic/Mild Disease (n=65) | 9.2% (6) | 1.4 (0.5-4.1) | 1.3 (0.4-3.9) |
| Moderate/Severe Disease (n= 30) | 15.2% (5) | 2.5 (0.8-7.9) | 2.5 (0.7-8.4) |
| **Unvaccinated population (N=711)** | | | |
| No infection (n=330) | 7.3% (24) | Reference | Reference |
| Asymptomatic/Mild Disease (n=261) | 6.9% (18) | 0.9 (0.5-1.8) | 0.9 (0.5-1.8) |
| Moderate/Severe Disease (n= 120) | 13.3% (16) | 2.0 (1.0-3.8)) | 2.1 (1.1-4.3) |

The logistic regression models include the same covariates as the models for the overall cohort. Vaccine status is unknown for 51 subjects (5%).
